# Genotype and diet shape resistance and tolerance across distinct phases of bacterial infection

**DOI:** 10.1186/1471-2148-14-56

**Published:** 2014-03-22

**Authors:** Virginia M Howick, Brian P Lazzaro

**Affiliations:** 1 Department of Entomology, Cornell University, Ithaca, NY, USA

**Keywords:** Tolerance, Resistance, Host-pathogen interactions, *Drosophila*, Genotype-by-environment interactions

## Abstract

**Background:**

Host defense against pathogenic infection is composed of resistance and tolerance. Resistance is the ability of the host to limit a pathogen burden, whereas tolerance is the ability to limit the deleterious effects of a given pathogen burden. This distinction recognizes that the fittest host does not necessarily have the most aggressive immune system, suggesting that host-pathogen co-evolution involves more than an escalating arms race between pathogen virulence factors and host antimicrobial activity. How a host balances resistance and tolerance and how this balance influences the evolution of host defense remains unanswered. In order to determine how genotype-by-diet interactions and evolutionary costs of each strategy may constrain the evolution of host defense, we measured survival, fecundity, and pathogen burden over five days in ten genotypes of *Drosophila melanogaster* reared on two diets and infected with the Gram-negative bacterial pathogen *Providencia rettgeri*.

**Results:**

We demonstrated two distinct phases of infection: an acute phase that consists of high mortality, low fecundity, and high pathogen loads, and a chronic phase where there was a substantial but stable pathogen load and mortality and fecundity returned to uninfected levels. We demonstrated genetic variation for resistance in both phases of infection, but found genetic variation for tolerance only in the acute phase. We found genotype-by-diet interactions for tolerance, especially in the acute phase, but genotype-by-diet interaction did not significantly shape resistance. We found a diet-dependent positive relationship between resistance and tolerance and a weak evolutionary cost of resistance, but did not detect any costs of tolerance.

**Conclusions:**

Existing models of tolerance and resistance are overly simplistic. Multi-phase infections such as that studied here are rarely considered, but we show important differences in determination and evolutionary constraints on tolerance and resistance over the two phases of infection. Our observation of genetic variation for tolerance is inconsistent with simple models that predict evolutionary fixation of tolerance alleles, and instead indicate that genetic variation for resistance and tolerance is likely to be maintained by non-independence between resistance and tolerance, condition-dependent evolutionary costs, and environmental heterogeneity.

## Background

A host can deal with an infection by directly limiting a pathogen burden (resistance) or by withstanding the negative consequences of the given burden (tolerance). Biomedically, tolerance implies that the host with the most aggressive immune system may not necessarily be the healthiest [1]. Evolutionarily, tolerance expands the range of strategies a host could employ to maximize fitness under infection conditions, and may impose evolutionary constraints, requiring the host to balance these potentially antagonistic strategies. From the pathogen’s perspective, tolerance may relax or remove selection imposed by the canonical immune system, potentially resulting in a neutral or positive effect on pathogen fitness [2-4].There is widespread evidence that tolerance is an important feature of host defense, but how this strategy evolves in concert with resistance and other life-history traits in animals, especially across variable environments, remains unknown.

From the host perspective, the superficial outcome of both resistance and tolerance can be the same: the retention of fitness. However, the long-term ecological and evolutionary dynamics are predicted to have very different outcomes. Importantly, resistance is expected to have a direct negative effect on pathogen fitness, whereas tolerance will have a neutral or positive effect [2-4]. This increase in pathogen fitness promoted by host tolerance could result in increased pathogen prevalence, which may increase the benefit of alleles that confer tolerance in the host, possibly offsetting any costs of those alleles. Oppositely, hosts with costly resistance alleles may reduce pathogen prevalence to levels where the resistant allele is no longer beneficial [2-5]. Theoretical work has shown that this negative feedback loop under a resistance strategy could result in the maintenance of polymorphism for resistance alleles, whereas the positive feedback loop created when a tolerance strategy is employed is more likely to result in the fixation of tolerance alleles in the population [3]. In this study, we measured genetic variation for both tolerance and resistance to empirically test this simple model. Our prediction was that we would find little genetic variation for tolerance if the simple positive feedback model is true, but that negative feedback loops would result in considerable variation for resistance.

While the predicted evolutionary outcomes of tolerance and resistance are very different, the natural dynamics are complicated by both internal constraints and external variability. The model proposed by Roy [3] assumes that resistance and tolerance alleles are independent, that there is only a single locus determining each strategy, and that each strategy exhibits the same evolutionary cost. The model also assumes a single, non-evolving, pathogen genotype and a constant environment. Theoretical work has relaxed some these simplifications [6-8], showing that variation in costs of the defense strategies could maintain variation in both tolerance and resistance, but these issues have not been empirically addressed in animals. We empirically address these complexities by quantifying costs, response to altered dietary environment, genotype-by-diet interactions, and the relationship between these two strategies.

Disentangling defense into separate components of resistance and tolerance, and understanding how these distinct strategies are related to each other, is crucial to understanding the evolution of host defense. However, first we must know how tolerance manifests and how it should be quantified. Empirical studies in animals have mainly focused on measures of health as estimates of tolerance, including survival [9], weight [10], and red blood cell count [11], whereas plant work has focused on more direct fitness estimates such as total fruit set [12] and seed production [13]. These different metrics of tolerance could have different evolutionary outcomes because they may differentially affect pathogen fitness [14] and may trade off with different aspects of host physiology. Statistically, tolerance has been defined as the slope of the line where the health or fitness estimate is plotted against pathogen burden. Three recent studies have explicitly tested for natural genetic variation for tolerance in animals using this framework. Two found evidence for variation in tolerance [11,15], whereas one did not [10].

Although tolerance in practical terms has been studied in agriculture for over 100 years [16,17], recent interest in evolutionary questions about tolerance in natural systems was stimulated by a study in morning glory that demonstrated a negative relationship between resistance and tolerance [18]. However, this negative relationship does not seem to be generalizable, and the current consensus in the plant literature leans towards a positive relationship between these two strategies, where a host allocates defense resources to both resistance and tolerance [19,20]. In animals, Räberg et al. [11] found a negative relationship between resistance (peak pathogen load) and tolerance (weight and red blood cells) in laboratory mice infected with *Plasmodium chabaudi*. Tolerance and resistance may be correlated because of pleiotropic mechanisms, linkage disequilibrium, or physiological constraints. Inconsistencies in the relationship between resistance and tolerance could result from system-specific mechanisms [1] and/or different definitions or metrics of tolerance and resistance.

Dietary environment and metabolic status can affect defense through both tolerance and resistance. Resistance is affected by dietary environment [21,22], and there is antagonism between defense and growth/tissue repair [23-26] as well as reproduction [27]. We hypothesized that a tradeoff between resistance and tolerance might be mediated by processes that are responsive to dietary environment, with the underlying assumption that tolerance strategies include tissue repair and growth, while resistance includes canonical immunity. Alternatively, a positive relationship between these two strategies would suggest that increased resource allocation to defense results in simultaneously improved resistance and tolerance, as would be expected if the underlying mechanisms of these strategies were overlapping. We predicted that dietary environment would alter both tolerance and resistance, but that the relationship between the two might change in a diet-dependent manner.

In the present study, we were able to identify genetic variation for both tolerance and resistance and show that maintenance of this polymorphism could result from a balanced optimum between these two strategies, shaped by evolutionary costs of defense and genotype-by-environment interactions. We hypothesized that dietary environment would alter the balance between tolerance and resistance, and that different environments may favor different defense strategies. We estimated tolerance both as functions of survival and fecundity under infection conditions to understand whether these are in fact different processes and distinct forms of tolerance. We followed these traits over time, developing a framework for defense that acknowledges that infection is not a binary state but a dynamic process consisting of multiple phases of infection. This study sheds light on the evolvability of tolerance and resistance, and how these distinct strategies may influence the evolution of host-pathogen interactions.

## Results

### Dynamics of *P. rettgeri* infection

**Figure 1:**
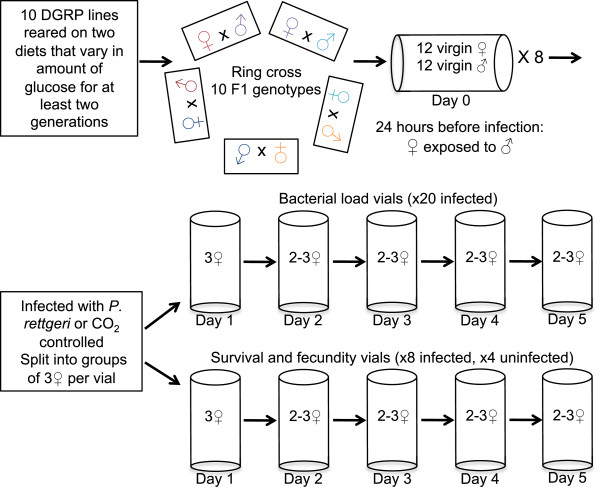
**Experimental design.** Outbred F1 progeny were derived from the ten DGRP lines using a ring crossing design, such that each line contributed a mother for one experimental genotype and a father for another. Virgin F1 females were exposed to males 24 hours prior to infection. Flies were infected with *P. rettgeri*. After infection flies were split into two groups: those for measuring bacterial load and those for measuring survival and fecundity. All vials were transferred daily and maintained at a density of 2–3 females. Each day a subsample of flies were removed for the destructive bacterial load assay. Daily mortality was recorded for the survival and fecundity vials, and the vials were saved to count the number of eventually emerging adult offspring. All of this was performed on the two diets that differed in the level of glucose.

**Figure 2:**
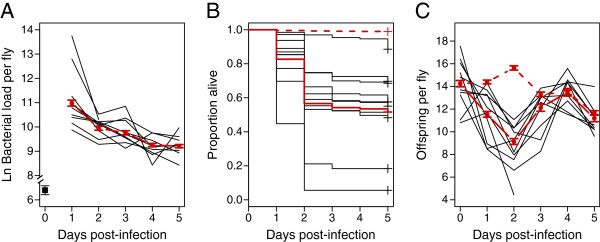
**Natural variation in defense.** Bacterial load **(A)**, survival **(B)**, and fecundity **(C)** over five days post-infection. The solid red line is the overall estimate of each phenotype calculated across all diets and genotypes. The black lines represent each genotype across both diets. The dashed red lines are the overall estimates of uninfected CO_2_ control flies. The majority of the mortality occurs within the first two days after infection, when bacterial loads are at their highest. We term this the “acute” phase of infection. Bacterial load and survival stabilize after day 3, into the “chronic” phase of infection. The difference in fecundity between infected and uninfected flies occurs during the acute phase, with a mean of 9.14 offspring per infected female and 15.63 offspring per uninfected female in the second day after infection. There is genetic variation for all three phenotypes measured.

In order to understand how defense dynamics change over time – including the relative balance of tolerance and resistance – we measured bacterial load, fecundity, and survival in ten outbred *Drosophila melanogaster* genotypes once per day for five days after infection with *Providencia rettgeri* (Figure 1). Each fly was infected with approximately 800 bacteria. Bacterial load increased to an average of approximately 5.9 × 10^4^ bacteria per fly in the first day after infection. Load decreased on Day 2 to 2.1 × 10^4^ bacteria per fly and did not significantly change between Days 2 and 3. By Day 4 average load levels decreased to 1.0 × 10^4^ bacteria per fly and did not significantly change between Days 4 and 5 (Figure 2a, Model F; Tables 1 and 2; Day: p < 0.001; Tukey’s test levels: Day 1: A; Day 2,3: B; Day 4,5: C). The majority of the mortality occurred within the first two days after infection; across all treatments, 56.6% of infected flies were dead by this point compared to 0.9% of the uninfected control flies. Between Days 3 and 5, survival of infected flies fell from 54.2% to 51.8% over all genotypes measured (Figure 2b, Model D, Table 3; Day: p < 0.001; Tukey’s test levels: Day 1: A; Day 2: B; Day 3: C; Day 4: D; Day 5: C, D). Fecundity per individual female was greatly reduced during the first three days of infection, the same time period when pathogen loads were highest and most mortality occurred. The largest difference between infected and uninfected occurred two days after infection, with a mean of 9.14 offspring per infected female and 15.63 offspring per uninfected female (Figure 2c, Model B, Table 4; Day: p < 0.001; Tukey’s test levels: Day 1: A; Day 2: B; Day 3: A,C; Day 4: C; Day 5: A). These infection dynamics revealed two distinct phases of infection, which has not been considered in previous theoretical or empirical work on tolerance. Based on this observation, we have split the infection into an “acute” phase and a “chronic” phase. The acute phase is defined as the first three days after infection, when pathogen levels are highest within the host and greatest mortality and reduction in fecundity occurs. The chronic phase is defined as Days 4 and 5, when pathogen levels are lower and appear stabilized and mortality and fecundity have returned to near uninfected levels.

**Table 1:** Possible terms included in regression analyses

**Factor**	**States**	**Effect type**	**Effect measured**	**Relevant models**
Genotype	10	Fixed or random	F1 cross genotype of flies	A,B,C,D,E,F
Diet	2	Fixed	Diet that flies were reared and phenotyped on	A,B,C,D,E,F
Load	Continuous		Daily bacterial load, natural log transformed	A,B,C,D
Day	5	Fixed	Day post-infection	A,B,C,D,E,F
Uninfected fecundity	Continuous		Daily fecundity of uninfected flies	A,B
Genotype*Diet	20	Fixed	Differential effect of diet on each genotype	A,C,E
Genotype*Load	Continuous		Differential effect of pathogen load on each genotype	A,C
Diet*Load	Continuous		Differential effect of pathogen load on each diet	A,B,C,D
Diet*Day	10	Fixed	Differential dynamics on each diet	B,D
Load*Day	Continuous		Differential dynamics of tolerance	B,D
Genotype*Diet*Load	Continuous		Differential effect of diet on tolerance	A,C
Diet*Load*Day	Continuous		Differential effect of diet on tolerance dynamics	B,D

*represents an interaction between factors.

See Methods for full models.

**Table 2:** Model F tested the effect of diet on resistance

**Factor**	**F-value**	**p-value**
Diet	10.53	0.001
Day	187.69	<0.001
Diet*Day	0.95	0.330

Bacterial load is the response variable. Genotype is a random factor in the model. Bacterial load changes over time and across dietary environment. Diet and Day post-infection are both predictors of Load.

*represents an interaction between factors.

**Table 3:** Model D tested the effect of diet on mortality tolerance

**Factor**	**z-value**	**Pr(<|z|)**
Load	6.33	<0.001
Diet	2.56	0.011
Day	6.58	<0.001
Diet*Load	-2.66	0.007
Load*Day	-6.06	<0.001
Diet*Day	-3.57	<0.001
Diet*Load*Day	3.78	<0.001

The proportion of flies alive each day post-infection is the response variable. Genotype is a random factor in the model. Load, Diet, and Day predict Mortality. Mortality tolerance is affected by Diet (Diet*Load), Day (Load*Day), and the interaction between these two factors (Diet*Load*Day).

**Table 4:** Model B tested the effect of diet on fecundity tolerance

**Factor**	**F-value**	**p-value**
Uninfected fecundity	3.12	0.078
Load	15.08	<0.001
Diet	35.1	<0.001
Day	44.22	<0.001
Load*Day	2.1	0.078
Diet*Load	0.14	0.709
Diet*Day	0.36	0.837
Load*Diet*Day	1.21	0.307

The response variable is Infected Fecundity. Genotype is a random factor in the model. Load, Diet, and Day predict Infected Fecundity. Diet does not affect fecundity tolerance (Diet*Load). Day post-infection marginally predicts fecundity tolerance (Load*Day).

### Is there genetic variation for tolerance and resistance?

After initial characterization of the dynamics of bacterial load, survival, and reproduction, we sought to determine whether the host *D. melanogaster* population was genetically variable for defense quality, and whether that defense quality varied with diet. The 10 F1 outbred genotypes in our study are derived from the *Drosophila* genetic reference panel (DGRP), a panel of 192 inbred lines that were each created from an independent single female collected in Raleigh, NC, USA [28]. To avoid effects of inbreeding, we chose 10 of these lines at random and established outbred F1 genotypes using a ring cross design, such that each of the 10 chosen DGRP lines contributed a mother to one experimental genotype and a father to another (Figure 1). All phenotypes were genetically polymorphic among our outbred progeny (Figure 2). Three linear models were fitted to the data to determine the magnitudes of genetic variation for resistance (systemic pathogen load), fecundity tolerance (the host’s ability to produce offspring given the pathogen burden), and mortality tolerance (the host’s ability to survive a given pathogen burden). Daily fecundity tolerance was estimated as the number of offspring produced each day normalized by the bacterial load estimated for the genotype on that day. Mortality tolerance was measured as the daily mortality rate per fly normalized to the bacterial load. The terms included in these models and their interpretations are given in Table 1. Separate models were run on the data from the acute and chronic phases of infection.

Genotype was a strong predictor of Bacterial Load in both the acute and chronic phase of infection (Model E; Table 5; Genotype: p < 0.001), clearly demonstrating genetic variation for resistance. We found genetic variation for fecundity tolerance in the acute phase of infection (Model A; Table 6; Genotype*Load: *p* < 0.001), but not in the chronic phase (Model A; Table 6; Genotype*Load: *p* = 0.155; Table 6). We also found genetic variation for mortality tolerance in the acute phase of infection (Model C; Table 7; Genotype*Load: *p* < 0.001), but not in the chronic phase (Model C; Table 7; Genotype*Load: *p* = 0.909). It is striking that Bacterial Load does not predict Infected Fecundity (Model A; Table 7; Load: *p* = 0.347) or Mortality (Model C; Table 7; Load: *p* = 0.413) in the chronic phase of the infection. Thus, although we have genetic variation for bacterial load in both the acute and chronic phase of infection, the impact of this burden on fitness differs dramatically between the two phases of infection.

**Table 5:** Model E tested for genetic variation in resistance

	**Days 1-3**		**Days 4-5**	
**Factor**	**F-value**	**P-value**	**F-value**	**P-value**
Genotype	15.37	<0.001	4.17	<0.001
Diet	5.09	0.024	1.72	0.19
Day	50.05	<0.001	0.43	0.512
Genotype*Diet	1.08	0.378	1.19	0.305

The response variable is Bacterial Load. The significant effect of Genotype represents genetic variation for resistance.

*represents an interaction between factors.

**Table 6:** Model A tested for genetic variation in fecundity tolerance

	**Days 1-3**		**Days 4-5**	
**Factor**	**F-value**	**p-value**	**F-value**	**p-value**
Uninfected fecundity	122.87	<0.001	70.39	<0.001
Load	16.05	<0.001	0.89	0.347
Genotype	8.74	<0.001	2.19	0.027
Diet	45.15	<0.001	9.52	0.002
Day	70.71	<0.001	11.02	<0.001
Genotype*Load	4.84	<0.001	1.50	0.155
Genotype*Diet	1.95	0.042	0.29	0.97
Diet*Load	0.003	0.954	0.01	0.922
Genotype*Diet*Load	2.95	0.002	2.01	0.044

The response variable is Infected Fecundity. The significant Genotype*Load interaction represents genetic variation for fecundity tolerance. The significant Genotype*Diet*Load interaction represents a genotype-by-environment effect for fecundity tolerance.

**Table 7:** Model C tested for genetic variation in mortality tolerance

	**Days 1-3**		**Days 4-5**	
**Factor**	**Deviance residuals**	**Pr(>Chi)**	**Deviance residuals**	**Pr(>Chi)**
Day	25.24	<0.001	4.58	0.032
Genotype	408.61	<0.001	17.22	0.045
Diet	11.25	<0.001	1.75	0.185
Load	12.49	<0.001	0.67	0.413
Genotype*Load	28.65	<0.001	4.04	0.909
Genotype*Diet	28.72	<0.001	18.37	0.019
Diet*Load	0.63	0.427	2.31	0.129
Genotype*Diet*Load	38.64	<0.001	5.05	0.752

The proportion of flies alive each day post-infection is the response variable. The significant Genotype*Load interaction represents genetic variation for mortality tolerance. The significant Genotype*Diet*Load interaction represents a genotype-by-environment effect for mortality tolerance.

### Complex relationship between defense strategies and diet

**Figure 3:**
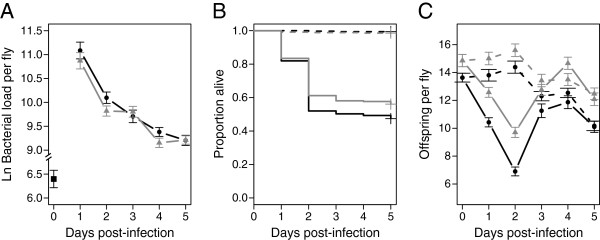
**Diet affects defense.** Bacterial load **(A)**, survival **(B)**, and fecundity **(C)** over five days post-infection across the two diets that vary in glucose concentration (black: high-sugar, 50 g/L; grey: low-sugar, 25 g/L). The dashed lines represent the uninfected CO_2_ control flies on each diet. Diet significantly affected all three phenotypes measured.

All phenotypes (survival, fecundity, bacterial load) varied significantly with diet (Figure 3). The 24-hour bacterial load measurement ranged from a median of 9.51 ln bacteria per fly to 15.22 ln bacteria per fly on the low-sugar diet (p < 0.001), and 9.18 to 14.99 ln bacteria per fly on the high-sugar diet (p < 0.001). Survival ranged from 8.33% to 88.9% on the low-sugar diet, and 2.82% to 88.2% on the high-sugar diet over five days after infection. The mean fecundity of infected flies at two days after infection ranged from 4.44 to 13.13 adult offspring per fly on the low-sugar diet (p < 0.001), and 2.52 to 9.19 (p < 0.001) on the high-sugar diet.

We hypothesized that the nutritional quality of the rearing diet might influence the quality of defense, either through resistance or tolerance. To test this, we evaluated defense quality after rearing on either high-sugar or low-sugar diets. Both Diet and Day post-infection were significant predictors of Load (Model F, Table 2; Diet: *p* = 0.0012, Day: *p* < 0.001), but there was no significant interaction between Diet and Day. This suggests that the dynamics of the resistance phenotype (*i.e.,* when the pathogen peaks, and how it is reduced in the host entering into the chronic phase) does not change across the diets used in this experiment (*p* = 0.330). Diet also significantly influenced mortality tolerance (Model D, Table 3; Diet*Load, *p* = 0.007), with higher mortality and thus lower tolerance observed on the high-sugar diet. In contrast, we found no evidence for a dietary effect on fecundity tolerance (Model B, Table 4; Bacterial load*Diet: *p* = 0.709). Thus, despite the fact that both fecundity and bacterial load are independently sensitive to diet, the relationship between them is not.

Given that diet shapes resistance and mortality tolerance, we hypothesized that the quantitative effects of diet depend on host genotype. We found evidence for genotype-by-diet determination of fecundity and mortality tolerance (Fecundity Tolerance: Model A, Table 6, Genotype*Diet*Load: *p* = 0.002; Mortality Tolerance, Model C, Table 7, Genotype*Diet*Load: *p* < 0.001) in the acute phase of infection. We also found a marginal genotype-by-diet effect on fecundity tolerance during the chronic phase of infection (Model A, Table 6; Genotype*Diet*Load: *p* = 0.044). This apparent interaction effect on fecundity tolerance disappears from the acute phase of the infection, if we reanalyze the data with per-fly daily fecundity estimated from the number of females that are alive at the end of each day instead of the number of flies that are alive at the beginning of the day (Additional file 1; Genotype*Diet*Load: acute: *p* = 0.390, chronic: *p* = 0.025). This indicates that the effect of the genotype-by-diet interaction on fecundity tolerance in the acute infection may be driven by the effect of the interaction on daily mortality. We did not find evidence for a genotype-by-diet interaction influencing resistance in either the acute or chronic phase of infection (Model E, Table 5; acute: *p* = 0.378, chronic: *p* = 0.305). Other experiments studying resistance against *P. rettgeri* with more genotypes and more extreme diets have found genotype-by-diet effects for resistance (unpublished results). This suggests our study may have lacked power to detect these effects, or that these effects are only detectable when the diets span a greater range of nutritional quality.

### Relationship between resistance and tolerance

**Figure 4:**
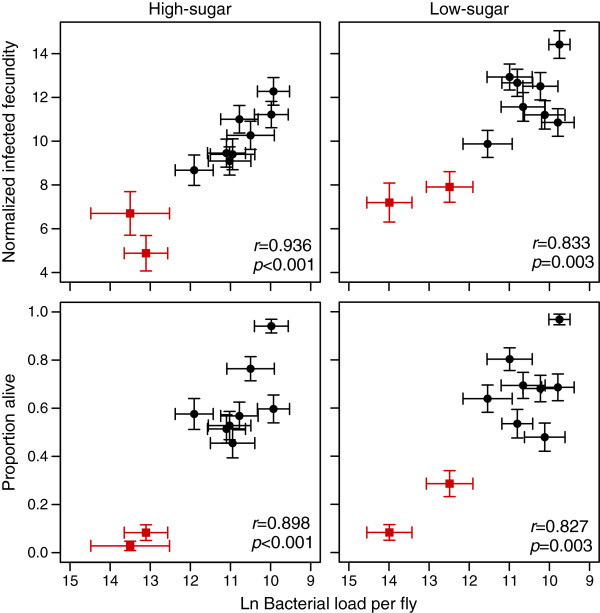
**Diet-dependent positive relationship between resistance and fecundity tolerance.** Each plotted data point represents a single genotype (error bars are standard errors). The square, red points represent the genotypes that experienced very high mortality after infection. A positive relationship was found between resistance, plotted as descending bacterial load 24 hours after infection, and normalized infected fecundity, estimated as the least-squares mean from Model A. When the two genotypes that had the highest mortality are excluded, the relationship is only significant on the high-sugar diet, suggesting that that these two traits can be decoupled in a diet-dependent manner. A similar pattern was seen when the proportion of flies surviving three days post-infection was used to estimate tolerance, except that the relationship was not significant on either diet when the high-mortality genotypes were excluded (see main text).

We evaluated the relationship between tolerance and resistance to determine whether these strategies trade off with one another, or if they act in an additive or synergistic manner. First, we defined overall fecundity tolerance as the least squares mean of infected fecundity from Model A using data from the acute phase of infection. This model incorporates uninfected fecundity to control for general vigor differences among the genotypes, as well as the bacterial load sustained by each genotype. Mortality tolerance was defined as the proportion of flies surviving three days post-infection (see Methods). Resistance is defined in this analysis as the inverse of systemic bacterial load at 24 hours after infection. Contrary to the expectation under tradeoff models, we found a positive correlation between resistance and fecundity tolerance on both diets when all genotypes are included (high sugar: *r* = 0.936, *p* < 0.001; low sugar: *r* = 0.833, *p* = 0.003, Figure 4). Interestingly, two genotypes that share a parental line and had less than 10% survival when infected (but 99% uninfected survival) as well as the highest bacterial load were entirely responsible for the positive relationship between fecundity tolerance and resistance on the low-sugar diet. When these two genotypes were removed from the analysis, the relationship remained significantly positive on the high-sugar diet (*r* = 0.890, *p* = 0.003), but becomes non-significant on the low-sugar diet (*r* = 0.394, *p* = 0.332). A similar pattern was seen when the proportion of flies surviving three days after infection was used to estimate tolerance, except that in this case, the relationship between resistance and tolerance became non-significant on both diets after excluding the two high-mortality genotypes (high sugar all genotypes: *r* = 0.898, *p* < 0.001; high sugar excluding two high-mortality genotypes *r* = 0.575, *p* =0.136; low-sugar including all genotypes: *r* = 0.827, *p* = 0.003; low-sugar exclude high-mortality genotypes: *r* = 0.251, *p* = 0.549). The stronger relationship on the high-sugar diet suggests that these traits can potentially be decoupled in a diet-dependent manner. Excluding the two high-mortality genotypes from this analysis may be biologically appropriate if the relationship between bacterial load and health or fitness is not linear. In other words, it is possible that at very high bacterial loads there is a threshold in which the host is not able to tolerate that burden, and health or fitness do not decrease at the same rate above this threshold as they do at lower bacterial loads.

**Figure 5:**
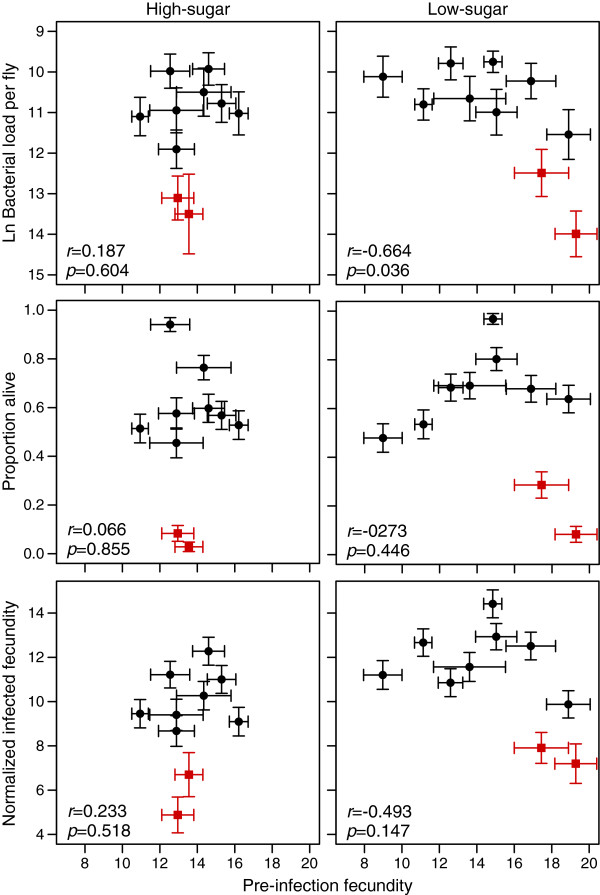
**Evolutionary costs of defense.** There is a weak diet-dependent evolutionary cost of resistance plotted as descending bacterial load 24 hours after infection and pre-infection fecundity. Pre-infection fecundity was estimated as the number of offspring per female produced during the 24 hours prior to infection. This relationship is dependent on the two high morality genotypes. Similar patterns are seen for the relationship between pre-infection fecundity and normalized infected fecundity as well as survival across both diets, but these were not significant.

The Roy [3] model assumes that the tolerance and resistance alleles are equally costly, and this cost is driving the feedback loops that will maintain variation for resistance but not for tolerance. To estimated the cost of each defense strategy, we tested the correlation of overall estimates of fecundity tolerance, mortality tolerance, and resistance with an overall measurement of uninfected fecundity, which was the sum of uninfected fecundity in the first three days after infection (see Methods). There was no relationship between daily-uninfected fecundity and any of the defense phenotypes (Additional file 2: Figure S1, inverse bacterial load *r* = -0.344, *p* = 0.3305; fecundity *r* = -0.324, *p* =0.361; mortality *r* = 0.553, *p* = 0.097). This provides no evidence for a fecundity cost of defense and indicates that general vigor did not drive defense quality in our study. We did find a weak negative correlation between fecundity prior to the infection experiment and bacterial load at 24 hours post-infection on the low-sugar diet (*r* = -0.664, *p* = 0.036, Figure 5). However, this tradeoff was not evident on the high-sugar diet (*r* = -0.187, *p* = 0.604; Figure 5) and became non-significant on the low-sugar diet if the genotypes displaying high mortality were excluded from the analysis. This trend is consistent with a diet-dependent tradeoff between reproduction and resistance in the early phase of an infection. Weak negative correlations were also seen between pre-infection fecundity and both fecundity and mortality tolerance on the low sugar diet, but none of these were significant (Figure 5). We found no correlation in quality of defense between the acute and chronic phases of the infection, reaffirming that these are truly independent infection states. The general lack of correlation between reproductive fitness and defense may be a result of low power of the experiments or unnaturally permissive experimental conditions.

## Discussion

We found two distinct phases of infection over the five days that defense was assessed. The acute phase (Days 1–3) was characterized by a high pathogen burden, severe mortality, and a reduction in fecundity. The chronic phase (Days 4–5) consisted of a still substantial but stable pathogen burden that was in no way predictive of mortality or fecundity. We found genetic variation for resistance, mortality tolerance, and fecundity tolerance in the acute phase of the infection, but only for resistance in the chronic phase. Experimental diet significantly influenced resistance and mortality tolerance during the acute phase of the infection. However, fecundity tolerance was not sensitive to diet. That is, even though diet altered both pathogen load and fecundity, it did so proportionally such that the relationship between load and fecundity (and hence fecundity tolerance) was unchanged. Using overall estimates of resistance and tolerance in the acute phase of infection, we found a positive relationship between resistance and tolerance, although the strength of this relationship was diet-dependent. We also demonstrate a weak evolutionary cost of resistance, but fail to detect any cost of tolerance.

During the chronic phase of infection, pathogen levels remained constant. This finding suggests either that the host and pathogen have reached an equilibrium where the immune system is killing bacteria at the same rate that the bacteria are replicating or that the bacteria have stopped replicating and are no longer exposed or sensitive to host immunity. We speculate that the latter may be occurring, because mortality and fecundity both return to near uninfected levels during the chronic phase. Further understanding the patterns of infection from the perspective of both host tolerance and pathogen virulence will clarify how both players interact to create host tolerance.

Current theoretical models describing variation of resistance and tolerance in the context of host-pathogen coevolution assume a single state of infection [2-4,6,7,14]. The empirical distinction between the acute and chronic phases of infection demonstrated here emphasizes the importance of looking at disease dynamics over time before extrapolating predictions about the spread of disease and evolution of defense in the wild. If transmission of *P. rettgeri* were to occur during the chronic phase of infection, then our results could be interpreted as supporting a model where tolerance alleles have fixed in the population. Infection tolerance through static chronic infection, which may not have major fitness consequences, should be integrated in future theoretical work on defense and infectious disease dynamics.

Consistent with previous studies (e.g., [29-31], we found genetic variation for resistance. We also found genetic variation for fecundity tolerance and for mortality tolerance. Previous studies in animals have found genetic variation in tolerance but see [10,11,15] and genetic variation for fecundity tolerance has been repeatedly observed in both the plant and animal literature [15,32,33], observations which we recapitulate in the acute (but not chronic) phase of infection. The continued presence of genetic variation for tolerance is inconsistent with basic theoretical models predicting tolerance alleles to be more likely to fix in the population than resistance alleles [3,4]. Non-independence between resistance and tolerance, or genotype-by-environment interactions may explain this inconsistency.

Although diet altered all three phenotypes measured (bacterial load, survival, and fecundity), it did not alter fecundity tolerance. In other words, the relationship between fecundity and pathogen load did not change across the two diets used in this experiment. However, mortality tolerance was very sensitive to diet. We expected that both fecundity and mortality tolerance would be sensitive to diet because both egg production and survival are regulated by metabolic pathways such as insulin-like signaling, which are responsive to alteration in dietary sugar (e.g. [34]). We observed that host females do not completely stop egg production under infection conditions, nor do we see any evidence for “egg dumping” – a rapid increase in egg output to maximize short-term fitness upon infection [35]. Instead, we observe only a minimal reduction in fecundity after infection, implying that females continue to invest in short-term reproductive output even though it may increase the risk of dying from infection and hence potentially limit lifetime fecundity.

We observed genotype-by-diet effects on mortality tolerance and fecundity tolerance, but saw no significant genotype-by-diet interaction for resistance. While increasing dietary glucose increased pathogen burden, there was little evidence for genetic variation in immunological sensitivity to dietary sugar in this study. Since there are significant genotype-by-diet effects on tolerance but not resistance during acute infection, we infer that the genes underlying tolerance are likely to function outside of the canonical resistance pathways.

In general, we found a positive relationship between resistance and tolerance, which supports the hypothesis that resource allocation to defense contributes to both resistance and tolerance [20]. Although, conceptually, a tradeoff between resistance and tolerance is easy to imagine and has some theoretical support ([13], but see [7,8,36,37]), the empirical evidence is very mixed. Several high-impact papers have demonstrated a negative relationship between the two strategies [11,12,18]. However, a meta-analysis of 31 ecological and agricultural studies of herbivore defense strategies did not find a significant relationship between resistance and tolerance [38], and several studies have demonstrated a positive relationship between the two strategies [39,40]. This lack of consensus may be a result of idiosyncrasies of specific host and pathogen combinations [41], as well as to employment of different measures of tolerance and resistance across studies. Our observation that the strength of the relationship between resistance and tolerance is diet-dependent suggests that environmental heterogeneity could decouple these two traits and allow them to evolve independently.

The observed tradeoff between pre-infection fecundity and bacterial load was driven by two genotypes that both were derived from line RAL-832. After the experiment was performed, it was discovered that this genotype contains a premature stop codon in the antimicrobial peptide gene, *Diptericin. Diptericin* is a downstream effector of the IMD pathway, which is responsible for resistance against Gram-negative pathogens. Although traditional life-history theory would suggest that an evolutionary tradeoff may be a result of limited resources between maintenance of the immune system and reproduction, we speculate from our results that it is more likely driven by direct costs of immunity, such as collateral damage from active antimicrobial activity. Other work has demonstrated a tradeoff between immunity and lifespan by showing that constitutive activation of the Gram-negative bacterial recognition protein, PGRP-LE, reduces lifespan. This phenotype can be rescued in flies with a homozygous loss-of-function mutation in the NF-kB transcription factor, Relish [42]. These results, combined with our study, suggest that the downstream effectors are the modulators of these tradeoffs, because if the tradeoff was simply based on resource availability, functional downstream effectors would play a smaller role than recognition and signaling molecules.

We did not observe any costs of tolerance. This suggests that resistance is more costly than tolerance, at least in terms of early-life reproduction. It is possible that under different conditions, increased power, or through measurements of different life-history traits, such as longevity, we may have found a cost of tolerance. Although this is the first attempt to identify costs of tolerance in animals as uninfected fitness, many studies in plants have found costs of tolerance [7,13], but some have failed to detect these costs [37,43,44].

We have empirically demonstrated that defense as tolerance and resistance is a dynamic process with multiple phases of infection, and that these defense strategies are differentially affected by diet and genotype-by-diet interactions. Future theoretical work should aim to incorporate these complexities into disease models, while empirical studies should acknowledge these alternative, yet synergistic, strategies and how they change over time and environment.

## Conclusion

Here we show that infection dynamics as well as dietary environment influence estimates of both tolerance and resistance, emphasizing that infection status is not a dichotomous state but a continuum that may yield different defense strategies in different contexts. The presence of genetic variation for fecundity and mortality tolerance is contrary to simple theoretical models, and we show that context dependence and non-independence between defense strategies may be maintaining this variation. The presence of a weak evolutionary cost of resistance, but no costs of tolerance suggests that direct costs of limiting a pathogen burden may be more costly than dealing with the negative consequences of that burden.

## Methods

### Drosophila stocks and diets

Ten lines were randomly chosen from the *Drosophila* Genetic Reference Panel (DGRP): RAL-26, RAL-217, RAL-272, RAL-318, RAL-370, RAL-374, RAL-595, RAL-732, RAL-832, RAL-897. The lines were collected from the Raleigh, NC, USA farmer’s market in 2003 and inbred with 20 generations of full-sib matings [28]. These lines represent a random snapshot of natural genetic variation. For at least two generations prior to the experiment, all lines were maintained on two diets that varied in the amount of D-glucose. Both diets contained 5% brewer’s yeast w/v, 1% agar, 0.035% phosphoric acid, and 0.42% propionic acid. The low sugar diet contained 2.5% D-glucose, and the high sugar diet contained 5% D-glucose.

### Generation of experimental flies

Outbred F1 progeny were derived from the ten DGRP lines using a ring crossing design, such that each line contributed a mother for one experimental genotype and a father for another (see Figure 1). The parental generation was maintained at a low density, approximately 20 females per bottle (30 ml food), and cleared after two days of egg laying to avoid overcrowding effects on offspring. Virgin experimental flies were aged for 3–5 days in groups of twelve flies per vial (8 ml food). For the 24-hour period before infection, females were exposed to males of the same genotype in groups of 12 females and 12 males per vial (Day 0 vial).

### Bacterial infection

The strain of *Providencia rettgeri* used in this experiment is a Gram-negative extracellular natural pathogen of *D. melanogaster*. It was isolated from the hemolymph of wild caught *D. melanogaster*[45]. Overnight cultures were started from a single bacterial colony and were grown overnight in liquid LB at 37°C with shaking. Prior to infections, the overnight culture was diluted in LB to an A_600_ of 0.2. Flies were infected by pricking in the thorax with a 0.15 mm dissecting pin (Fine Science Tools) dipped in the diluted overnight culture of *P. rettgeri*. This method delivered approximately 800 bacteria to each fly. Flies were anesthetized on CO_2_ for 3–4 minutes during infection. Control anesthetized flies were held on CO_2_ for the same amount of time. Infections were performed over two days for each replicate. The genotype order of infections was determined using a random number generator, with 5 genotypes infected on each of the two days. The infected females were placed in groups of three females per vial and the males were discarded. Approximately 96 females were infected per genotype per diet per experimental replicate.

### Post-infection fly handling

Female flies were placed in a fresh ‘Day 1′ vial in groups of three immediately after infection or control handling. The flies were assigned either for measurement of bacterial load or for measurement of fecundity and survival. For each genotype on each diet, there were four uninfected survival/fecundity vials, eight infected survival/fecundity vials and approximately 20 bacterial load vials. For the next five days after infection, all flies were transferred each day and maintained at a density of two to three female flies per vial. The number of surviving flies was recorded daily as the flies in the survival/fecundity treatment were transferred to new vials. These vials were retained and emerging adult offspring were counted for up to 16 days after the transfer. The number of emerged adults was used as the measure of fecundity.

### Bacterial load assay

Bacterial load was estimated for each genotype on each diet once a day for five days after infection. Individual females were placed in 500 μl LB and homogenized with a sterile pestle. On Day 1, the homogenates were diluted 1:100 dilution prior to plating. On Days 2 and 3, homogenates were diluted 1:10. No dilution was necessary on Days 4 and 5. These homogenates were then plated onto LB agar using a WASP 2 spiral plater (Microbiology International, Bethesda, MD, USA). Plates were grown overnight at 37°C and resulting colonies were counted using a ProtoCOL plate counting system (Microbiology International) to estimate the number of viable bacteria infecting each fly.

### Statistical analyses

All statistical analyses were performed in R [46]. Mixed-effect models were analyzed using the nlme package [47]. Separate generalized linear models were built for analysis of mortality tolerance, fecundity tolerance, and resistance. The possible factors included in the models were Genotype, Diet, Day Post-Infection, Bacterial Load, and Uninfected Fecundity. Genotype was considered a fixed effect when the biological question related to the existence of genetic variation for a given trait, but genotype was considered a random effect when questions were asked at a species level (*e.g*. Does diet affect tolerance?). Genotype was used as a random effect in these models, because the question of how diet affects defense treats genotype as a random draw from a population. Linear contrasts were performed using the multcomp package in R [48]. The Tukey’s test was used to compare load, fecundity, and mortality rate for each day. For these comparisons, interaction terms were not included in the models.

Two models were tested to estimate fecundity tolerance. Model A tested for the presence of natural genetic variation in tolerance. The response variable is the infected fecundity corrected for the number of females put into the vial. In order to normalize the distribution of the residuals, all fecundity values were taken to the power of 0.6, which was chosen using the boxcox function in the MASS package in R [49]. The Box-Cox procedure identifies an appropriate exponent to use to transform the data to a normal distribution by calculating the log-likelihood of the data over a range of power transformations. Uninfected Fecundity was included in the models to account for variation in general vigor. Genetic variation for tolerance is indicated by a significant Genotype-by-Load interaction, which shows that certain genotypes are better able to handle the severity of an infection than others. A significant three-way interaction between Genotype, Load, and Diet represents a genotype-by-environment effect on tolerance. For this analysis, the data were split into the acute and chronic phase of infection. Day 1–3 is considered acute phase because that is where most of the mortality and the peak pathogen intensity occur. Day 4–5 is considered the chronic phase of infection because the negative impacts of infection are not as extreme. Model B tested for the effect of diet and day on fecundity tolerance. To correct for correlations that result from repeated measurements on the same group of flies, first-order autoregressive error structure was fit to the model using the corAR1() function from the R package lme4 [50].

Model A evaluated genetic variation in fecundity tolerance:

$$\begin{array}{ll} \mathrm{Infected}\;\mathrm{Fecundity}= & \mathrm{Uninfected}\;\mathrm{Fecundity}+\mathrm{Load}\\ & +\mathrm{Genotyp}e_{\mathrm{fixed}}+\mathrm{Diet}+\mathrm{Day}+\\ & \mathrm{Genotype}*\mathrm{Load}+\mathrm{Genotype}\\ & *\mathrm{Diet}+\mathrm{Load}*\mathrm{Diet}\\ & +\mathrm{Genotype}*\mathrm{Load}*\mathrm{Diet}+\mathrm{error} \end{array}$$

Model B tested the effect of diet on fecundity tolerance and whether tolerance changed over days of the experiment:

$$\begin{array}{ll} \mathrm{Infected}\;\mathrm{Fecundity}= & \mathrm{Uninfected}\;\mathrm{Fecundity}+\mathrm{Load}\\ & +\mathrm{Genotyp}e_{\mathrm{random}}+\mathrm{Diet}+\mathrm{Day}\\ & +\mathrm{Load}*\mathrm{Diet}+\mathrm{Day}*\mathrm{Load}+\mathrm{Day}\\ & *\mathrm{Diet}+\mathrm{Load}*\mathrm{Diet}*\mathrm{Day}+\mathrm{corAR}1 \end{array}$$

Mortality tolerance was estimated using generalized linear models that followed a binomial distribution and used the number of dead and alive flies as the response variables for each experimental replicate.

Model C tested for genetic variation in mortality tolerance:

$$\begin{array}{l} \mathrm{Probability}\;\mathrm{of}\;\mathrm{mortality}\;\left(\mathrm{number}\;\mathrm{dead},\mathrm{number}\;\mathrm{alive}\right)\\ \;=\mathrm{Load}+\mathrm{Genotyp}e_{\mathrm{fixed}}+\mathrm{Diet}+\mathrm{Day}\;+\mathrm{Genotype}\\ \;*\mathrm{Load}+\mathrm{Genotype}*\mathrm{Diet}+\mathrm{CFU}*\mathrm{Diet}\\ \;+\mathrm{Genotype}*\mathrm{Diet}*\mathrm{Load}+\mathrm{error} \end{array}$$

Model D tested the effect of Diet on mortality tolerance:

$$\begin{array}{l} \mathrm{Probability}\;\mathrm{of}\;\mathrm{morality}\;\left(\mathrm{number}\;\mathrm{dead},\mathrm{number}\;\mathrm{alive}\right)\\ \;=\mathrm{Load}+\mathrm{Genotyp}e_{\mathrm{random}}+\mathrm{Diet}+\mathrm{Day}\;\\ \;+\mathrm{Genotype}*\mathrm{Load}+\mathrm{Genotype}*\mathrm{Diet}\\ \;+\mathrm{Load}*\mathrm{Diet}+\mathrm{Genotype}*\mathrm{Diet}*\mathrm{Load}+\mathrm{error} \end{array}$$

Model E tested for genetic variation and a genotype-by-environment effect on resistance, and Model F considered the effects of Diet and Day post-infection on resistance. The response variable in these models was the natural log of the estimated bacterial load.

Model E tested genetic variation in resistance:

$$\mathrm{Load}=\mathrm{Genotyp}e_{\mathrm{fixed}}+\mathrm{Diet}+\mathrm{Day}+\mathrm{Genotype}*\mathrm{Diet}+\mathrm{error}$$

Model F tested the effect of Diet on resistance:

$$\mathrm{Load}=\mathrm{Genotyp}e_{\mathrm{random}}+\mathrm{Diet}+\mathrm{Day}+\mathrm{Genotype}*\mathrm{Diet}+\mathrm{error}$$

Correlations between resistance and mortality tolerance or fecundity tolerance were estimated on each day and diet, as well as for an overall estimate for each phenotype determined from the acute stage of the infection (Days 1–3). For this latter correlation, the maximum bacterial load measurement for each genotype was used as the overall estimate of resistance. Fecundity tolerance estimates were least squares mean of infected fecundity standardized by uninfected fecundity and the median bacterial load as determined from Model A. Mortality tolerance was estimated as the proportion of flies surviving three days after infection. Overall estimates of tolerance were estimated from similar models that included all data from Days 1–3.

## Abbreviations

DGRP: *Drosophila* genetic reference panel.

## Competing interests

The authors declare that they have no competing interests.

## Authors’ contributions

VMH and BPL designed the experiments. VMH performed the experiments. VMH analyzed the data. VMH and BPL wrote the manuscript. Both authors read and approved the final manuscript.

## Supplementary Material

Additional file 1: Table S1 and Model S1 Model S1 tests for genetic variation in fecundity tolerance. The response variable (Infected Fecundity) is corrected by the number of females that were alive at the end of the 24-hour period. The results from this model are displayed in **Table S1**. Click here for file

Additional file 2: Figure S1 No detected cost of defense as uninfected fecundity. Cost of defense measured as the sum of uninfected fecundity (CO_2_ control females) in the first three days after CO_2_ exposure correlated with proportion alive three days post infection, normalized infected fecundity in the acute phase of the infection, and bacterial load at 24 hours after infection on the low- and high-sugar diets. The square, red points represent the genotypes that experienced very high mortality after infection. None of these correlations were significant. Click here for file
