# Supplementary material for: Genotype and diet shape resistance and tolerance across distinct phases of bacterial infection
Source: BMC Evol Biol. 2014 Mar 22;14:56. doi: 10.1186/1471-2148-14-56 (PMC3997931; doi:10.1186/1471-2148-14-56)
Supplement: Additional file 1: Table S1 and Model S1 — Model S1 tests for genetic variation in fecundity tolerance. The response variable (Infected Fecundity) is corrected by the number of females that were alive at the end of the 24-hour period. The results from this model are displayed in Table S1. [file 1471-2148-14-56-S1.docx]

**Additional File 1**

**Model S1**

Model S1 evaluated genetic variation in fecundity tolerance. The response variable, Infected Fecundity, was corrected by the number of females that were alive at the end of the 24-hour period of egg-laying in each vial instead of the number of females that entered the vial alive.

Infected Fecundity (exit) = Uninfected Fecundity + Load + Genotype _fixed_ + Diet + Day + Genotype*Load + Genotype*Diet + Load*Diet + Genotype*Load*Diet + error

| **Table S1** | Days 1-3 | |  | Days 4-5 | |
| --- | --- | --- | --- | --- | --- |
| **Factor** | **F-value** | **p-value** |  | **F-value** | **p-value** |
| Uninfected Fecundity | 122.92 | <0.001 |  | 62.6 | <0.001 |
| Load | 7.02 | 0.008 |  | 0.83 | 0.362 |
| Genotype | 5.61 | <0.001 |  | 2.62 | 0.008 |
| Diet | 21.11 | <0.001 |  | 4.31 | 0.038 |
| Day | 16.75 | <0.001 |  | 5.54 | 0.019 |
| Genotype*Load | 3.55 | <0.001 |  | 1.44 | 0.176 |
| Genotype*Diet | 1.98 | 0.038 |  | 0.42 | 0.912 |
| Diet*Load | 0.2 | 0.658 |  | 0.49 | 0.484 |
| Genotype*Diet*Load | 1.06 | 0.389 |  | 1.94 | 0.052 |

**Table S1 Model S1 tests for genetic variation in fecundity tolerance**

The response variable is Infected Fecundity corrected by the number of females that were alive at the end of the 24-hour period. The significant Genotype*Load interaction represents genetic variation for fecundity tolerance. The significant Genotype*Diet*Load interaction represents a genotype-by-environment effect for fecundity tolerance.
