# Supplementary material for: Genotype and diet shape resistance and tolerance across distinct phases of bacterial infection
Source: BMC Evol Biol. 2014 Mar 22;14:56. doi: 10.1186/1471-2148-14-56 (PMC3997931; doi:10.1186/1471-2148-14-56)
Supplement: Additional file 2: Figure S1 — No detected cost of defense as uninfected fecundity. Cost of defense measured as the sum of uninfected fecundity (CO2 control females) in the first three days after CO2 exposure correlated with proportion alive three days post infection, normalized infected fecundity in the acute phase of the infection, and bacterial load at 24 hours after infection on the low- and high-sugar diets. The square, red points represent the genotypes that experienced very high mortality after infection. None of these correlations were significant. [file 1471-2148-14-56-S2.pdf]

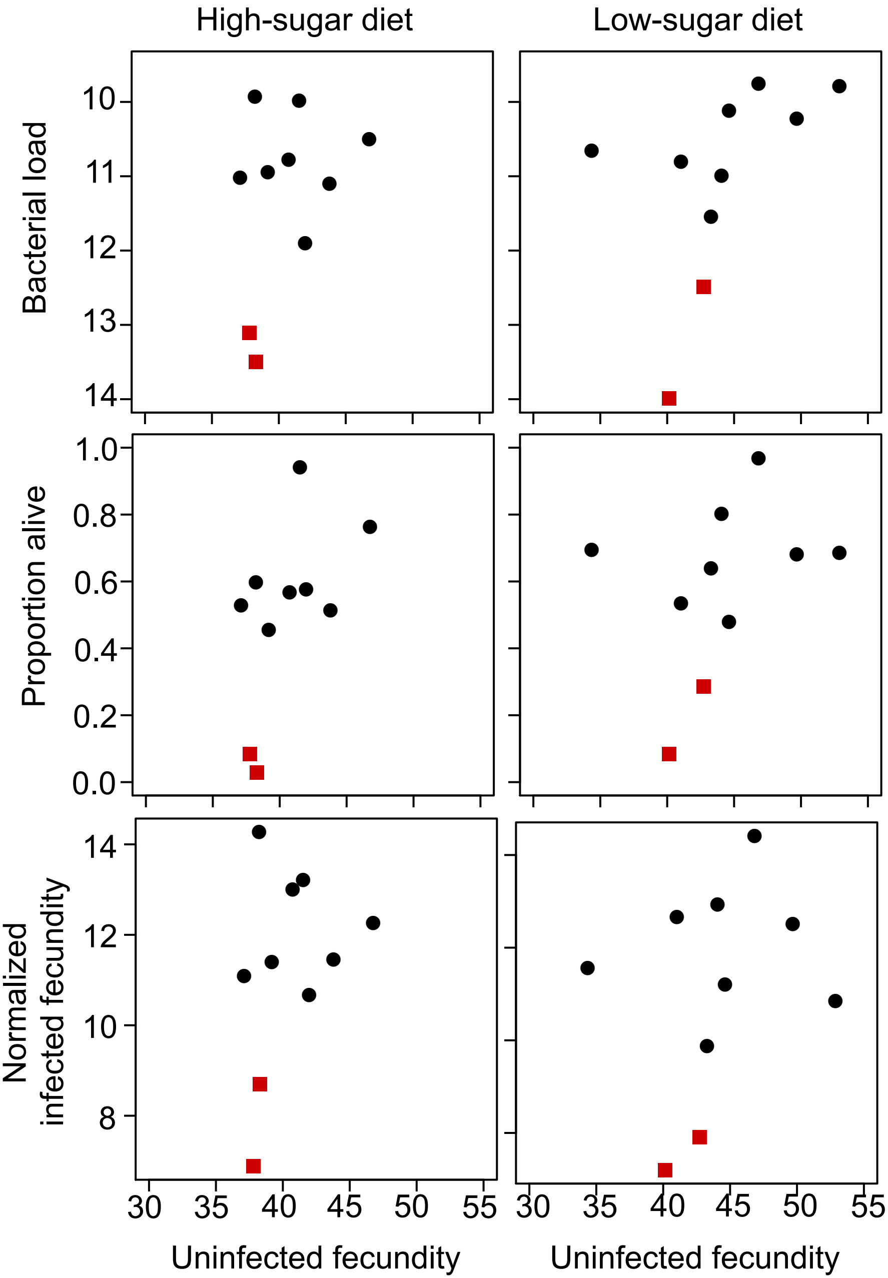

High-sugar diet
Low-sugar diet
Bacterial load
10
11
12
13
14
Proportion alive
1.0
0.8
0.6
0.4
0.2
0.0
Normalized
infected fecundity
14
12
10
8
30
35
40
45
50
55
Uninfected fecundity
Uninfected fecundity
